# Supplementary material for: Study of entanglement via a multi-agent dynamical quantum game
Source: PLoS One. 2023 Jan 27;18(1):e0280798. doi: 10.1371/journal.pone.0280798 (PMC9882909; doi:10.1371/journal.pone.0280798)
Supplement: S1 Appendix — (PDF) [file pone.0280798.s001.pdf]

Supplementary Material for "Study of entanglement via a multi-agent dynamical quantum game"

Proof of Theorem 1

Let us write explicitly the correlation matrix for n species, including a special, $(n+1)$th, specie that employs two observables. Thus,

$$C = \begin{bmatrix} M(n,p,\rho) & \Gamma \\ \Gamma^T & D \end{bmatrix} \tag{1}$$

Here, the $n \times n$ sub-matrix, $M(n,p,\rho) = I_{n\times n} + \rho A(n,p)$, where $A(n,p)$ is the adjacency matrix of $G(n,p)$. In addition,

$$\Gamma^T = \begin{bmatrix} \rho^0_{1,n+1} & \cdots & \rho^0_{n,n+1} \\ \rho^1_{1,n+1} & \cdots & \rho^1_{n,n+1} \end{bmatrix}, \tag{2}$$

and

$$D = \begin{bmatrix} 1 & \eta \\ \eta & 1 \end{bmatrix} \tag{3}$$

where it is assumed, without loss of generality, that η is real. In what follows it is assumed that ρ is non-negative.

As C is a correlation matrix it must be positive semidefinite. The block decomposition of C allows for an equivalent requirement,

$$M(n,p,\rho) \succ 0, \quad D - \Gamma^T M(n,p,\rho)^{-1}\Gamma \succeq 0 \tag{4}$$

known as Schur complement condition for positive semidefiniteness.

As C is a random matrix there should be some distribution of the largest possible ρ admitting the conditions (4). A known result in random matrix theory, based on concentration of measure arguments, has it that the distribution of the eigenvalues of $A(n,p)/\sqrt{np}$ approaches the Wigner semicircle distribution with radius 2 in the $n \to \infty$ limit (see Theorem 2.8.1 in [1], and Exercise 3.1 in [2]),

$$\text{Prob}\left(\lambda\left(\frac{A(n,p)}{\sqrt{np}}\right) \leq x\right) \longrightarrow \text{Wsc}(x)$$

Therefore, for sufficiently large n it follows that

$$\lambda\left(M(n,p,\rho)^{-1}\right) = (1+\gamma\rho\sqrt{np})^{-1} \tag{5}$$

where $\gamma \sim \text{Wsc}(\gamma)$. Furthermore, that $M(n,p,\rho) \succ 0$ means $\rho < -1/(\gamma\sqrt{np}) = \mathcal{O}(1/\sqrt{np})$, for $\gamma < 0$ and sufficiently large n.

The additional condition in (4) further restricts the bound on ρ. To find out how we shall express the distribution of ρ^*, the maximum value of ρ, such that (4) hold.

From (4) and (5) it follows that for large n,

$$D - \frac{\Gamma^T \Gamma}{1 + \gamma\rho\sqrt{np}} = D - \frac{np\rho^2(1+\delta)}{1 + \gamma\rho\sqrt{np}} \begin{bmatrix} 1 & p \\ p & 1 \end{bmatrix} \succeq 0 \tag{6}$$

where δ is a discrete random variable satisfying $\delta \le (n - \lfloor np \rfloor)/\lfloor np \rfloor \simeq (1-p)/p$. The expression in the numerator on the right follows from known concentration inequalities concerning sums of Bernoulli random variables; Thus, for large n, the sum $\sum_{i=1}^{n} (\rho_{i,n+1}^{j})^2$ centers around $nE[(\rho_{i,n+1}^{j})^2] = np\rho^2$, and, similarly, $\sum_{i=1}^{n} \rho_{i,n+1}^{0} \rho_{i,n+1}^{1}$ centers around $nE[\rho_{i,n+1}^{0} \rho_{i,n+1}^{1}] = np^2\rho^2$. The random variable δ accounts for the uncertainty in these estimates.

The right hand side in (6) is equivalent to non-negativity of the trace and determinant of the underlying matrix difference. This leads to,

$$\frac{np\rho^2(1+\delta)}{1 + |\gamma|\,\rho\sqrt{np}} \le g(\eta, p) \tag{7}$$

where $g(\eta, p)$ the same one defined in the theorem. In other words,

$$(\rho\sqrt{np})^2 - |\gamma|\, g'(\eta, p, \delta)(\rho\sqrt{np}) - g'(\eta, p, \delta) \le 0 \tag{8}$$

where $g'(\eta, p, \delta) = g(\eta, p)/(1+\delta)$. Solving for $\rho\sqrt{np}$ yields,

$$\rho\sqrt{np} \le \frac{1}{2}\left(|\gamma|\, g'(\eta, p, \delta) + \sqrt{\gamma^2 g'(\eta, p, \delta)^2 + 4g'(\eta, p, \delta)}\right) \le |\gamma|\, g'(\eta, p, \delta) + 2\sqrt{g'(\eta, p, \delta)} \tag{9}$$

We thus may identify the right hand side above as the random variable $\rho^*\sqrt{np}$. Therefore,

$$\frac{\rho^*\sqrt{np}}{g'(\eta, p, \delta)} = |\gamma| + \frac{2}{\sqrt{g'(\eta, p, \delta)}} \tag{10}$$

which holds for sufficiently large n.

Because γ is distributed according to $\mathrm{Wsc}(\gamma)$ in the $n \to \infty$ limit, and similarly $|\gamma|$ is distributed according to $\mathrm{Wsc}_+(|\gamma|)$, it follows from (10) that

$$\mathrm{Prob}\left(\frac{\rho^*\sqrt{np}}{g(\eta, p)} \le \underbrace{\frac{\nu}{1+\delta} + 2\sqrt{\frac{1+\delta}{g(\eta, p)}}}_{\zeta} \,\middle|\, \delta \right) = \mathrm{Prob}\left(\frac{\rho^*\sqrt{np}}{g'(\eta, p, \delta)} \le \nu + \frac{2}{\sqrt{g'(\eta, p, \delta)}} \,\middle|\, \delta \right) \longrightarrow \mathrm{Wsc}_+(\nu) \tag{11}$$

where $\nu \in [0, 2]$. That is,

$$\mathrm{Prob}\left(\frac{\rho^*\sqrt{np}}{g(\eta, p)} \le \zeta \,\middle|\, \delta \right) \longrightarrow \mathrm{Wsc}_+\left((1+\delta)\zeta - 2\frac{(1+\delta)^{3/2}}{\sqrt{g(\eta, p)}} \right) \tag{12}$$

Note that by the very definitions of ζ and $g(\eta,p)$, the product $\zeta g(\eta,p) = \mathcal{O}(\sqrt{1-\eta})$. Finally, in the $n\to\infty$ limit,

$$\mathrm{Prob}\left(\frac{\rho^*\sqrt{np}}{g(\eta,p)}\le\zeta\right) = E_\delta\left[\mathrm{Prob}\left(\frac{\rho^*\sqrt{np}}{g(\eta,p)}\le\zeta\,\middle|\,\delta\right)\right] \longrightarrow E_\delta\left[\mathrm{Wsc}_+\left((1+\delta)\zeta - 2\frac{(1+\delta)^{3/2}}{\sqrt{g(\eta,p)}}\right)\right] \tag{13}$$

from which the theorem follows.

Nonlocality and dynamics

Linearization

Locally, the system may be described by its Lyapunov exponents, the eigenvalues of $\exp(Jt)$ with the Jacobian J, where

$$J = \begin{bmatrix} \gamma - \sum_{i=1}^n B_i v_i & -B_1 c & \cdots & -B_n c \\ B_1 v_1 & -\zeta_1 + B_1 c & & 0 \\ \vdots & & \ddots & \\ B_n v_n & 0 & & -\zeta_n + B_n c \end{bmatrix}. \tag{14}$$

Let us assume that $\forall i\in[n], \zeta_i=\zeta, v_i = v$; and also $\forall i \in \{2,\ldots,n\}, B_i = B$. Note that B_1 may be different than the others! Rewriting the Jacobian:

$$J = \begin{bmatrix} \gamma - B_1 v - (n-1)Bv & -B_1 c & -Bc & \cdots & -Bc \\ B_1 v & -\zeta + B_1 c & 0 & \cdots & 0 \\ Bv & 0 & -\zeta + Bc & \ddots & \vdots \\ \vdots & \vdots & \ddots & \ddots & 0 \\ Bv & 0 & \cdots & 0 & -\zeta + Bc \end{bmatrix} \tag{15}$$

The characteristic polynomial of J:

$$\begin{aligned} p_J(\lambda) &= (\gamma - [(n-1)B + B_1]v - \lambda)(-\zeta + Bc - \lambda)^{n-1}(-\zeta + B_1 c - \lambda) + \\ &\quad + B_1^2 cv(-\zeta + Bc - \lambda)^{n-1} + (n-1)B^2 cv(-\zeta + B_1 c - \lambda)(-\zeta + Bc - \lambda)^{n-2} = \\ &= (-\zeta + Bc - \lambda)^{n-2}(\gamma - [(n-1)B + B_1]v - \lambda)(-\zeta + Bc - \lambda)(-\zeta + B_1 c - \lambda) + \\ &\quad + (-\zeta + Bc - \lambda)^{n-2} B_1^2 cv(-\zeta + Bc - \lambda) + (-\zeta + Bc - \lambda)^{n-2}(n-1)B^2 cv(-\zeta + B_1 c - \lambda) \end{aligned} \tag{16}$$

Which implies that $\lambda_{mul} = Bc - \zeta$ appears as an eigenvalue with algebraic multiplicity of (at least) $n-2$. λ_{mul} corresponds to dynamical modes for which c, v_1 and $\sum_{k=2}^n v_k$ are constant; i.e., the only dynamics are amongst the $n-1$ "homogeneous" virus species, and therefore not particularly interesting for our purposes.

The other three eigenvalues are the solutions of the cubic equation:

$$\begin{aligned}&(\gamma - [(n-1)B + B_1]\, v - \lambda)\,(-\zeta + Bc - \lambda)\,(-\zeta + B_1 c - \lambda) + \\ &+ B_1^2 cv\,(-\zeta + Bc - \lambda) + (n-1)B^2 cv\,(-\zeta + B_1 c - \lambda) = 0.\end{aligned} \tag{17}$$

We shall examine the dynamics of the system near a point where the normalized populations are all 1. Moreover, we are only interested in cases where the viruses all have positive population payoffs, i.e. $B_1, B > 0$. First, let us rewrite (17) using the variable $\tilde{\lambda} := \lambda + \zeta$:

$$\begin{aligned}&\left(\gamma + \zeta - [(n-1)B + B_1]\, v - \tilde{\lambda}\right)\left(Bc - \tilde{\lambda}\right)\left(B_1 c - \tilde{\lambda}\right) + \\ &+ B_1^2 cv\left(Bc - \tilde{\lambda}\right) + (n-1)B^2 cv\left(B_1 c - \tilde{\lambda}\right) = 0.\end{aligned} \tag{18}$$

Now, we substitute $B_1 = \frac{\beta}{\sqrt{2}}\cos\theta$, $B = \frac{\beta}{\sqrt{2(n-1)}}\sin\theta$, $c = v = 1$, and also define $\delta := \gamma + \zeta$:

$$\begin{aligned}&\left(\delta - \sqrt{\frac{n-1}{2}}\beta\sin\theta - \frac{\beta}{\sqrt{2}}\cos\theta - \tilde{\lambda}\right)\left(\frac{\beta}{\sqrt{2(n-1)}}\sin\theta - \tilde{\lambda}\right)\left(\frac{\beta}{\sqrt{2}}\cos\theta - \tilde{\lambda}\right) + \\ &+ \frac{\beta^2}{2}\cos^2\theta\left(\frac{\beta}{\sqrt{2(n-1)}}\sin\theta - \tilde{\lambda}\right) + \frac{\beta^2}{2}\sin^2\theta\left(\frac{\beta}{\sqrt{2}}\cos\theta - \tilde{\lambda}\right) = 0.\end{aligned} \tag{19}$$

Generally, given parameters $\beta, \gamma, \zeta, \theta$, one may find some value $n_c = n(\beta, \gamma, \zeta, \theta)$, such that for any $n \le n_c$ the system would admit at least one non-negative Lyapunov exponent (note for some range of the parameters we would have $n_c = 0$). This function $n(\beta, \gamma, \zeta, \theta)$ is given implicitly by considering $\Re\left(\tilde{\lambda}_{\max}\right) = \zeta$, where $\tilde{\lambda}_{\max}$ is the solution to (19) having the largest real part.

Non-entangled case

In the non-entangled (equally-correlated) case, we assume $\mathscr{B}_{\mathcal{CV}_k} = 2\sqrt{2/n} =: \mathscr{B}$ for all k, implying $B_1 = B = \beta/\sqrt{2n}$. We may define a new dynamic variable: $\bar{v} \triangleq \left(\sum_{i=1}^{n} v_i\right)/n$, and replace our system of $n+1$ equations with only two:

$$\begin{aligned}\dot{c} &= \gamma c - Bcn\bar{v} \\ \dot{\bar{v}} &= -\zeta\bar{v} + Bc\bar{v}.\end{aligned} \tag{20}$$

This system admits the following equilibrium point:

$$\bar{v} = \frac{\sqrt{2}\gamma}{\sqrt{n}\beta}, \qquad c = \sqrt{2n}\zeta/\beta \tag{21}$$

and the following Jacobian:

$$J_{NE} = \begin{bmatrix}\gamma - Bn\bar{v} & -Bcn \\ B\bar{v} & -\zeta + Bc\end{bmatrix} \tag{22}$$

Let us find its eigenvalues. The characteristic polynomial is:

$$\begin{aligned}\det\left(J_{NE}-\lambda I\right) &= \left(\gamma - Bn\bar{v} - \lambda\right)\left(-\zeta + Bc - \lambda\right) + B^2 cn\bar{v} = \\ &= \lambda^2 + \left(-\gamma + Bn\bar{v} + \zeta - Bc\right)\lambda - \gamma\zeta + \gamma Bc + \zeta Bn\bar{v}. \end{aligned} \tag{23}$$

Let us use the notation $\delta = \gamma + \zeta$ and assume $c \approx 1$, $v_{tot} \approx 1$:

$$\begin{aligned}\lambda_{\pm} &= \frac{1}{2}\left[\gamma - \zeta + B\left(c - n\bar{v}\right)\right] \pm \frac{1}{2}\left(\left[\gamma - \zeta + B\left(c - n\bar{v}\right)\right]^2 - 4\left(-\gamma\zeta + \gamma Bc + \zeta Bn\bar{v}\right)\right)^{1/2} \approx \\ &\approx \frac{1}{2}\left[\gamma - \zeta - \frac{\beta}{\sqrt{2n}}\left(n-1\right)\right] \pm \frac{1}{2}\left(\left[\gamma - \zeta - \frac{\beta}{\sqrt{2n}}\left(n-1\right)\right]^2 - 4\left(-\gamma\zeta + \frac{\beta\gamma}{\sqrt{2n}} + \frac{\beta\zeta n}{\sqrt{2n}}\right)\right)^{1/2} = \\ &= \frac{1}{2}\left[\gamma - -\frac{\zeta\beta}{\sqrt{2n}}\left(n-1\right)\right] \pm \frac{1}{2}\left[\delta^2 - \frac{2\gamma\beta}{\sqrt{2n}}\left(n+1\right) - \frac{2\zeta\beta}{\sqrt{2n}} + \frac{\beta^2}{2n}\left(n-1\right)^2\right]^{1/2}. \end{aligned} \tag{24}$$

The discriminant is:

$$\Delta = \delta^2 - 2B\left(n+1\right)\delta + B^2\left(n-1\right)^2 = \delta^2 - \sqrt{2}\beta\delta\left(\sqrt{n} + \frac{1}{\sqrt{n}}\right) + \frac{\beta^2}{2}\left(n - 2 + \frac{1}{n}\right), \tag{25}$$

which vanishes for $\delta_{\pm} = \frac{\beta(n+1)}{\sqrt{2n}} \pm \sqrt{2}\beta$. For large enough values of n we have:

$$\Delta \approx \frac{\beta^2 n}{2}\left[1 - 2\sqrt{\frac{2}{n}}\frac{\delta}{\beta} + \frac{2\left(\delta^2 - \beta^2\right)}{\beta^2 n}\right].$$

Thus, it follows that $\lambda_{+} \approx -\zeta + \frac{\delta^2}{2\sqrt{2n}\beta}$ and $\lambda_{-} \approx \gamma - \beta\sqrt{\frac{n}{2}} + \frac{2\beta^2 - \delta^2}{2\sqrt{2n}\beta} \approx \gamma - 2\beta/\mathscr{B}$. Now we shall find the eigenvectors:

$$\begin{aligned} J_{NE} - \lambda_{+} I &= \begin{bmatrix} \delta - \beta\sqrt{\frac{n}{2}} - \frac{\delta^2}{2\sqrt{2n}\beta} & -\beta\sqrt{\frac{n}{2}} \\ \frac{\beta}{\sqrt{2n}} & \frac{2\beta^2 - \delta^2}{2\sqrt{2n}\beta} \end{bmatrix} + O\left(\frac{1}{n}\right), \\ J_{NE} - \lambda_{-} I &= \begin{bmatrix} \frac{\delta^2 - 2\beta^2}{2\sqrt{2n}\beta} & -\beta\sqrt{\frac{n}{2}} \\ \frac{\beta}{\sqrt{2n}} & -\delta + \beta\sqrt{\frac{n}{2}} + \frac{\delta^2}{2\sqrt{2n}\beta} \end{bmatrix} + O\left(\frac{1}{n}\right). \end{aligned} \tag{26}$$

Thus we have:

$$\boldsymbol{u}_{+} \approx \begin{bmatrix} \frac{\delta^2 - 2\beta^2}{2\beta^2} \\ 1 \end{bmatrix}, \qquad \boldsymbol{u}_{-} \approx \begin{bmatrix} 1 \\ \frac{\delta^2 - 2\beta^2}{2n\beta^2} \end{bmatrix}. \tag{27}$$

Maximally-entangled case

In the maximally-entangled case, $\mathscr{B}_{\mathcal{CV}_1} = 2\sqrt{2}$ and for any $k > 1$, $\mathscr{B}_{\mathcal{CV}_k} = 0$. Thus, $B_1 = \beta/\sqrt{2}$ and $B = 0$. The system admits the following equilibrium point:

$$c = \sqrt{2}\zeta/\beta, \qquad v_1 = \sqrt{2}\gamma/\beta, \qquad v_k = 0\ \forall k > 1. \tag{28}$$

The eigenvalues are $\lambda_{mul} = -\zeta$ with algebraic multiplicity $n-2$, and the other three are solutions of the following cubic equation:

$$\begin{aligned} &(\gamma - B_1 v - \lambda)(-\zeta - \lambda)(-\zeta + B_1 v - \lambda) + B_1^2 cv(-\zeta - \lambda) = 0 \\ \iff &(\lambda + \zeta)\left[(\gamma - B_1 v - \lambda)(-\zeta + B_1 v - \lambda) + B_1^2 cv\right] = 0. \end{aligned} \tag{29}$$

So we see that $\lambda = -\zeta$ actually has algebraic multiplicity $n-1$, and the remaining two eigenvectors are the roots of:

$$\begin{aligned} &(\gamma - B_1 v - \lambda)(-\zeta + B_1 v - \lambda) + B_1^2 cv = 0 \\ \iff &\lambda^2 - \lambda(\gamma - \zeta) - \gamma\zeta + \gamma B_1 v + \zeta B_1 v - B_1^2 v^2 + B_1^2 cv = 0 \\ \iff &\lambda_\pm = \frac{1}{2}(\gamma - \zeta) \pm \frac{1}{2}\left[(\gamma - \zeta)^2 - 4\left(-\gamma\zeta + \gamma B_1 v + \zeta B_1 v - B_1^2 v^2 + B_1^2 cv\right)\right]^{1/2} = \\ &= \frac{1}{2}(\gamma - \zeta) \pm \frac{1}{2}\left[(\gamma - \zeta)^2 - 4\left(-\gamma\zeta + \beta\gamma v/\sqrt{2} + \beta\zeta v/\sqrt{2} - \beta^2 v^2/2 + \beta^2 cv/2\right)\right]^{1/2}. \end{aligned} \tag{30}$$

Let us assume that $v \approx c \approx 1$. Using these approximations, the Jacobian has the form:

$$J = \begin{bmatrix} \gamma - B_1 & -B_1 & 0 & \cdots & 0 \\ B_1 & -\zeta + B_1 & 0 & \cdots & 0 \\ 0 & 0 & -\zeta & \ddots & \vdots \\ \vdots & \vdots & \ddots & \ddots & 0 \\ 0 & 0 & \cdots & 0 & -\zeta \end{bmatrix} \tag{31}$$

and the remaining two eigenvalues are:

$$\begin{aligned} \lambda_\pm &= \frac{1}{2}(\gamma - \zeta) \pm \frac{1}{2}\left[(\gamma - \zeta)^2 - 4(-\gamma\zeta + \gamma B_1 + \zeta B_1)\right]^{1/2} = \\ &= \frac{1}{2}(\gamma - \zeta) \pm \frac{1}{2}\left[(\gamma + \zeta)(\gamma + \zeta - 4B_1)\right]^{1/2} = \frac{1}{2}(\gamma - \zeta) \pm \frac{1}{2}\left[(\gamma + \zeta)\left(\gamma + \zeta - 2\sqrt{2}\beta\right)\right]^{1/2} = \\ &= \frac{1}{2}(\gamma - \zeta) \pm \frac{1}{2}\left[\delta\left(\delta - 2\sqrt{2}\beta\right)\right]^{1/2} \end{aligned} \tag{32}$$

where we have used the notation $\delta = \gamma + \zeta$. The corresponding eigenvectors are:

$$\boldsymbol{u}_{\pm} = \begin{bmatrix} 2B_1 \\ \delta - 2B_1 \mp \sqrt{\delta\left(\delta - 2\sqrt{2}\beta\right)} \\ 0 \\ \vdots \\ 0 \end{bmatrix}. \tag{33}$$

And for λ_{mul} we have the eigenvectors $\{\boldsymbol{e}_j\}_{j=2}^{n}$, where $\boldsymbol{e}_j$ is the vector with jth entry 1 and all other entries 0 (note the indices start from 0).

We must consider three cases: $\delta = 4B_1$, $\delta < 4B_1$ and $\delta > 4B_1$ (recall that $B_1 = \beta/\sqrt{2}$). We start with the former.

1. $\gamma + \zeta = 4B_1 = 2\sqrt{2}\beta$: we obtain the eigenvalue $\lambda_2 = (\gamma - \zeta)/2$ with algebraic multiplicity 2. Let us express it with β rather than ζ:

$$\lambda_2 = \frac{\gamma - \zeta}{2} = \frac{\gamma + \gamma - 2\sqrt{2}\beta}{2} = \gamma - \sqrt{2}\beta = \gamma - 4\beta/\mathscr{B}_{\mathcal{CV}_1}, \tag{34}$$

while the eigenvalue λ_{mul}, with multiplicity $n-1$, obtains the form:

$$\lambda_{mul} = -\zeta = \gamma - 2\sqrt{2}\beta = \gamma - 8\beta/\mathscr{B}_{\mathcal{CV}_1}. \tag{35}$$

Compare with the Lyapunov exponents of the non-entangled case: $\lambda_- = \gamma - \beta\sqrt{n/2} + O\left(1/\sqrt{n}\right) \approx \gamma - 2\beta/\mathscr{B}$ and $\lambda_+ = -\zeta + O\left(1/\sqrt{n}\right) \approx \gamma - 2\sqrt{2}\beta$. Interestingly, for $n = 4$ the cells have the same Lyapunov exponents in the non-entangled and maximally-entangled states, up to terms in the order of $1/\sqrt{n}$.

2. $\gamma + \zeta < 4B_1$: Let us denote $\Delta \triangleq \sqrt{(\gamma+\zeta)\left(4B_1 - \gamma - \zeta\right)}$; thus, we obtain $\lambda_{\pm} = \frac{1}{2}\left(\gamma - \zeta \pm i\Delta\right)$.

3. $\gamma + \zeta > 4B_1$: For the case of $\gamma + \zeta > 4B_1$, we denote $\Delta \triangleq \sqrt{(\gamma+\zeta)\left(\gamma + \zeta - 4B_1\right)}$; thus, we obtain $\lambda_{\pm} = \frac{1}{2}\left(\gamma - \zeta \pm \Delta\right)$.

---

[1] T. Tao, *Topics in random matrix theory.* American Mathematical Society, 2012.
[2] C. Bordenave, "Lecture notes on random matrix theory," 2019.
