# Supplementary material for: Study of entanglement via a multi-agent dynamical quantum game
Source: PLoS One. 2023 Jan 27;18(1):e0280798. doi: 10.1371/journal.pone.0280798 (PMC9882909; doi:10.1371/journal.pone.0280798)
Supplement: Attachment — Submitted filename: Response_Letter_180822.docx [file pone.0280798.s002.docx]

August 19, 2022

Dear Editor,

We wish to thank you for considering our manuscript for publication in *PLOS ONE*. We also thank the reviewers for their remarks. Please find below our point-by-point response to all comments raised by the reviewers, as well as our revised manuscript (both marked-up and unmarked versions), which was further clarified and broadened thanks to these comments.

Additionally, we wish to clarify that all data required to reproduce the results of our manuscript is contained within the manuscript itself and the supporting information file provided as part of this submission. Also, we do not wish to amend our funding statement.

Many thanks and best wishes,

Prof. Avishy Carmi (on behalf of all authors)

Reviewer #1

My main concern regarding this study is that it is unclear to me how in the manuscript that the authors make the jump from having ligands and cell receptors to presenting a “quantum mechanical description” of the payoff matrix without giving proper justification for the existence of such a matrix.

We are thankful to the Reviewer for these remarks. As the manuscript suggests, the premise of this work may be understood as considering, solely within the realm of quantum information, a certain type of enhancement of the Bell-CHSH game. In our enhanced game, the original Bell-CHSH game is played on many instances by a large number of entities (*pieces*) that comprise two or more *factions* (where each faction corresponds to a distinct “species”). All entities belonging to the same faction employ the exact same strategy, and the rules of the game are defined to be such that any *mini-game* results in one additional piece being added to the winning faction, and one piece being deducted from the losing faction. Perhaps it is more convenient to consider the enhanced game as a game played by *n+1* players - i.e., each player “controls” a single faction by choosing its strategy.
While this artificial construction is not likely to describe what occurs in an actual ecological system, the game is intuitively explained by terms belonging to the field of population dynamics, and is studied by borrowing a few methods from game theory and population dynamics. Therefore, the biological terms “cell”, “virus”, “receptor” and “ligand” should not be taken seriously - they merely form a way of giving the game a “livelier” description, just like the names “king”, “knight”, “bishop” etc. given to chess pieces. From a game-theoretic perspective, the essence of the game is completely detached from any medieval or biological imagery; it lies solely in the rules of the game. In our case, the rules for the mini-games are the same as for the Bell-CHSH game. The payoff matrix comprises a way of describing both the rules for the mini-games, and their effect on the enhanced game.
Note that the payoff matrix is also completely unrelated to anything quantum-mechanical. Indeed, the payoff matrix represents the rules of the mini game itself, while the quantum mechanical description appears because we wish the factions to employ a *quantum strategy* for the game. There is nothing quantum about the payoff matrix itself, and it would have been the same if the factions were to utilize only classical strategies for the game. A paragraph that clarifies these points was added to the manuscript.

To my understanding, the authors are making the implicit assumption that the receptor-ligand couple is able to be measured in any arbitrary basis, when in fact, no basis other than the computational one is given to be corresponding to an observable in their description. One might define probabilistic states, however, it is unclear to me how one might formulate these on a Hilbert space.

A crucial point to note is that the random variables C and V, denoting the cell’s receptor and the virus’ ligand respectively, are *classical* binary random variables. These random variables do not span any Hilbert space. As stated above, the quantum mechanical description arises only because we assume that the cells and viruses employ a quantum strategy for this game.
The quantum state then describes an “external” quantum system employed by the players (the factions) solely for the sake of implementing a quantum strategy. This is perhaps the most “artificial” part of our construction – indeed, the qubits are not even assumed to be physically contained within the actual biological bodies of the cells and viruses. To some extent they cannot be, as the quantum state is assumed to be a “global” two-qubit state, which determines the strategy for all cells and viruses simultaneously. To understand how this all fits together, let us write down explicitly the rules of the game (assuming that the factions wish to use quantum strategies):

1. The game is played by *n+1* players. Out of those, *n* are designated as “predator-type” players, and the remaining one is designated to be of “prey-type”.
2. Setup:
   a. The referee prepares a quantum state consisting of *n+1* qubits. The state can be thought of as being chosen by the referee. The referee then assigns each qubit to a single player.
   b. Each player choses two single-qubit operators, denoted by ${\hat{\mathcal{O}}}_{C}^{0}$ and ${\hat{\mathcal{O}}}_{C}^{1}$ for the “prey-type” player and by ${\hat{\mathcal{O}}}_{V_{k}}^{0}$ and ${\hat{\mathcal{O}}}_{V_{k}}^{1}$ for the *k*-th “predator-type” player. Those operators must have only $\pm1$ as their eigenvalues. The chosen operators completely determine the strategies; in fact, after this step, the players have no effect on the game whatsoever. They may just sit back and observe how the game plays out.
   c. The referee prepares the “game board”, which is a system (or simulation of a system) that consists of 2*N* pieces for each player, placed randomly and uniformly within some closed environment. The pieces may be considered as colored in different colors representing the players they belong to. Out of those *2N* pieces of the same color, *N* carry a note that says “0”, and the other *N* carry a note that says “1”.
3. Now the game begins, and the pieces start to travel the board at random. With every time-step (some arbitrarily small $\Delta t$) that follows, the referee places more pieces for the prey-type player (at random positions on the board) and eliminates random pieces for the predator-type players. The number of pieces born and eliminated respectively at each time step is determined by the parameters $\gamma,\zeta_{k}$. When a new piece gets placed on the board, its note is chosen at random by tossing a fair coin.
4. Whenever a piece that belongs to the prey-type player meets a piece belonging to one of the predator-type players (say the *k*-th one), they play a single mini-game, which proceeds as follows:
   a. The referee checks the two interacting pieces for their notes. She writes down $a$ for the bit that was written down on the prey-type player’s piece, and $b$ for the bit from the predator-type player’s piece.
   b. The referee performs a measurement on the quantum state. She measures ${\hat{\mathcal{O}}}_{C}^{a}$ on the prey-type player’s qubit, ${\hat{\mathcal{O}}}_{V_{k}}^{b}$ on the *k­-*th predator-type player’s qubit, and the identity operator $I$ on all the other predator-type players’ qubits.
   c. The outcomes of the measurements on the prey-type player’s qubit and the *k*-th predator-type player’s qubit are denoted $C$ and $V$, respectively. The referee then computes the *predator payoff*, using the formula $P=\left( -1 \right)^{ab}\cdot C\cdot V$
   d. If $P=1$, the referee removes the prey-type player’s piece off the board and replaces it by a new piece that belongs to the *k*-th predator-type player. If $P=-1$, the referee removes the predator-type player’s piece and replaces it by a new prey-type player piece. The bit-valued note for the new piece is chosen by tossing a fair coin.
   e. The mini-game has now ended. The referee resets the quantum system to its original state, and the game proceeds as before.
5. A player that loses all their pieces is eliminated from the game. The goal is to survive for as long as possible.

This extended description of the game was added to the manuscript, as well as several further clarifications.

To be clear, they should be able to provide an explanation for the minus-test, i.e., what is the difference between the state they define in (8) and its orthogonal state. If these two states cannot be distinguished in their observational configuration, the authors cannot argue for the existence of such pure states, they can only define them as mixed states, which contain classical correlations and not quantum ones.

Our elaborate response above to the previous point should clarify this confusion as well.

The authors argue that the parameters they are utilizing (i.e. θa and φb) can be defined in the range of [0, π], when in fact these parameters can only take certain discrete values, to be consistent with the description they provide in section 2.1. The authors define a and b as the physiological states, and treat them as inputs for their operators. Hence, the reason how parameters θa and φb are able to take continuous values which can range between 0 and π is unclear to me, and I believe it needs further explanation.

Indeed, each one of the indices a,b takes two values, 0 and 1. Thus θ_a denotes a pair of angles, θ_0 and θ_1. These two angles can be chosen independently, and each one can take an arbitrary value within the range of [0, π]. However, from Eq. (12) it is clear that an optimal strategy (in the sense of maximizing the Bell-CHSH parameter) must choose these two angles to be π/2 radians apart (of course, the same is true for φ_b). Indeed, such an optimal choice is written down in Eq. (14). Note that, like many other details of our results, this constraint is inherently embedded in the proposed game, in the sense that it follows from the construction of our game along with the assumption that the predator-type player’s strategy is optimal. Of course, we do not make any claims of generality here; indeed, one could conceive of many other “predator-prey quantum games’’ for which the dynamics and optimal strategies would be immensely different. Our paper merely presents one example of such a game.
The angles θ_a were renamed α_a, to avoid confusion with the unrelated angle θ defined in section 2.6.

To sum up, the authors need to clarify, in much simpler terms without mathematical obfuscation, the connection between their configuration and its quantum mechanical description. Sadly, in its current form, the paper seems like two different papers of lower qualities, one describing a Lotka-Volterra system for a population of cell-virus couples, and another providing a review of Bell’s theorem with some additional demonstration of population dynamics. I don’t see the justifications or explicit assumptions that tie these two together. If there are, they are either too implicit, omitted or not thought out by the authors. For this work to be published, I believe the authors should very clearly identify and make explicit that which assumptions they are proposing to argue that a multi-agent dynamical game with discrete inputs, and indistinguishable/probabilistic descriptions of states can be formulated by a “quantum mechanical description”.

These concerns have been addressed in the first point (see above). We clarify again that the game presented in the paper is explicitly designed such that the “score” of each player is the size of their faction’s population, and that the time evolution of these scores is described by the Lotka-Volterra equations. Moreover, the gameplay consists of many mini-games which are instances of the Bell-CHSH game, in which the players utilize a quantum strategy determined by a single “global” quantum state and a pair of operators for each player. The state and operators are both determined in the setup phase of the game.

Following the suggestion above, we have now clearly indicated the assumptions behind the proposed quantum game and their justification in a new subsection, titled “The rules of the game – a summary”.

Reviewer #2

The authors study a predator-prey system that has been contrived to have correlation dynamics given by the CHSH operator. It displays quantum features such monogamy of entanglement between a cell interacting with different viruses. This is a work of art. It's not conceivable that this models any realistic cell-virus interactions. I have no idea why the authors would have wanted to study this, but since PLOS ONE does not evaluate significance, I'm not going to reject based on this. The criterion is technical correctness, and the manuscript is correct, the authors are clearly familiar with nonlocality and entanglement theory and have applied it correctly. The Lotka-Volterra dynamics are not easy to calculate, and the authors did a good job with them. The concerns about availability of data do not apply, since there's no experimental data to report; the results shown in Fig. 3 and Fig. 4 can be reproduced by the reader by re-running the simulation.

We are very thankful to the Reviewer for these remarks, as well as for the positive assessment.
