# Supplementary material for: Study of entanglement via a multi-agent dynamical quantum game
Source: PLoS One. 2023 Jan 27;18(1):e0280798. doi: 10.1371/journal.pone.0280798 (PMC9882909; doi:10.1371/journal.pone.0280798)
Supplement: Attachment — Submitted filename: Response_Letter_031222.docx [file pone.0280798.s003.docx]

December 4, 2022

Dear Editor,

We wish to thank you for considering our manuscript for publication in *PLOS ONE*. We also thank the reviewers for their remarks. Please find below our point-by-point response to all comments raised by the reviewers, as well as our revised manuscript (both marked-up and unmarked versions), which was further clarified and broadened thanks to these comments. We feel that the main concerns at this point stem from a mere confusion we might have created (regretfully) in our previous response to the reviewers and from the intuitive analogy we chose. An effort was made now in order to prevent such confusion among future readers.

Many thanks and best wishes,

Prof. Avishy Carmi (on behalf of all authors)

Reviewer #1

I would like to thank the authors for their detailed elaboration of their arguments and I believe the paper is in a much better state right now, however, I still have some serious objections and concerns that I think the authors should further clarify before publication.

We thank the reviewer for their additional comments.

1- In their response, the authors note “A crucial point to note is that the random variables C and V, denoting the cell’s receptor and the virus’ ligand respectively, are classical binary random variables.”, however, in the revised version of the article on lines 208-209 they wrote “if the cell and virus’ choices for C and V are allowed to be taken as quantum measurement outcomes over a shared entangled state, then the Bell-CHSH parameter may go up to a maximum of 2√ 2”. This is of course possible, however, I fail to see the distinction of this setup and a classical joint probability distribution with hidden variables (especially since C and V are defined as strictly classical binary random variables). The argument of non-locality and ‘quantum-ness’ here needs further clarification.

The point to be taken here is that the single cell-virus interactions are modeled to behave just as the original Bell-CHSH game. Perhaps the comment “A crucial point to note is that the random variables C and V, denoting the cell’s receptor and the virus’ ligand respectively, are classical binary random variables” was confusing (it does not appear on the manuscript itself, only on the previous response letter). It was written in response to the following remark by the Reviewer: “the authors are making the implicit assumption that the receptor-ligand couple is able to be measured in any arbitrary basis, when in fact, no basis other than the computational one is given to be corresponding to an observable in their description”. Then let us further clarify our response to this point:

There seemed to be some confusion regarding the separation between the game and its strategies. There is nothing quantum or classical about the receptor-ligand couple. They do not span a Hilbert space and are not “measured” in any basis. The receptor and ligand obtain values $C,V\in\left\{ \pm1 \right\}$, which are analogous to Alice and Bob’s $\pm1$ outputs in the original formulation of the Bell-CHSH game. Those are features of **the** **game**, not its (classical or quantum) **strategies**. Thus, $C$ and $V$ are neither classical nor quantum.

However, the process that produces the correlations $\rho_{ab}$ – the strategy – may be either classical or quantum (or hypothetically even “super-quantum” – e.g. Popescu-Rohrlich boxes, but such models are not considered in the manuscript). In particular, if a quantum strategy is employed, then the receptor-ligand correlations ($\rho_{ab}$ in the manuscript) may obtain values that cannot be explained using local hidden variable theories. Again, this just means that $C$ and $V$ behave like the $\pm1$ outputs provided by Alice and Bob in each round of the original Bell-CHSH game.

We hope that this explanation clarifies the reviewer’s concerns regarding this point. Moreover, the distinction between the game and its strategies is already made more explicit in the previous version of the manuscript; and additional explanation regarding the Bell-CHSH game was added to the section “Quantum strategies”. Thus, we hope that readers who have not been exposed to the response letters or the earlier versions of the manuscript would not be confused by this point.

2- I think the authors’ description of the relationship between their model and any actual biological system given between lines 134-152 is good, however, it definitely needs to be strongly reiterated in the discussion section. In its current form, sentences such as “For example, if we wish for the cell population (or its respective equivalent in the actual setup) to be immune to malevolent viruses, one could consider adding a new type of “virtual” (harmless) virus, and entangling it with the cells to the maximal extent possible; then, monogamy of entanglement would disallow any interactions between the malevolent viruses and the cells.” (given on lines 528-532) wildly misrepresents the extent of the authors’ work in this paper and may lead to serious misunderstanding.

We thank the reviewer for this important comment. The discussion section has been edited accordingly, removing any claim that can be misunderstood in such a manner.

3- I know the authors already cite the Eisert paper, but still I believe in the Quantum Strategies section it would be beneficial if they can provide (in plain terms and for a general audience) a discussion on what is the difference between a quantum strategy and a classical strategy employing hidden variables.

A new paragraph, further explaining the meaning of Bell’s theorem and its bearings on quantum vs. classical strategies, was added to this section.

4- The authors finish their paper with the sentence “…could one somehow use entanglement as a catalyst?” when in fact there is a literature on resource theory of entanglement for chemical reactions in the field of quantum thermodynamics, going even beyond entanglement and describing resources such as quantum discord (a non-local type of correlation that is not entanglement). If the authors wish to introduce this concept, I would recommend them to at least cite some recent papers on it (you may check these two papers by the team of Vlatko Vedral <https://journals.aps.org/pre/abstract/10.1103/PhysRevE.86.031922> - <https://royalsocietypublishing.org/doi/10.1098/rspa.2018.0037> and this relatively recent review article <https://www.sciencedirect.com/science/article/pii/S0303264716302799>).

The final paragraph was edited accordingly, and the citations were added.

I believe that, it is our duty as scientists to not mislead the audiences, especially on papers containing sensitive keywords such as viruses in the post-Covid reality that we are living in. I don’t think the authors would enjoy being cited at a popular science article that is claiming we can cull the next pandemic via entangling viruses with harmless ones (also I don’t think PONE editors would be much happy about it either, and I certainly don’t want to be the reviewer that played a part in it). Therefore, I urge the authors to rephrase their arguments related to the viruses and entanglement, avoid making any strong claims (such as the one on lines 528-532). And I recommend the editors to not go through with the publication before such measures are taken on the side of the authors.

We wholeheartedly agree with these concerns. These strong claims, that indeed may be misinterpreted, were removed from the manuscript.

Finally, there is a typo at line 262 where “prey-typ” is written instead of “prey-type.”

The typo has been corrected.

Reviewer #2

The authors are now explicitly they are studying an abstract quantum game, and that their paper shouldn't be understood as modelling virus-cell interactions. This was mostly done in response to the concerns of Referee #1, not mine, but nevertheless I think this improved the manuscript. I don't have any concerns and recommend publication.

However, the editor explicitly said that the code used for doing the simulation must be released, and the authors haven't done so.

We are very thankful to the Reviewer for the positive assessment. The code used for doing the simulation has now been made available online in: <https://github.com/smitke6/Predator_Prey/blob/79eed137918bab894e274ca14858a018dd893698/boide6.m>
